# Systemic gas embolism in three cases of CT-guided percutaneous lung puncture under general anesthesia: case reports and literature review

**DOI:** 10.3389/fmed.2026.1739881

**Published:** 2026-02-19

**Authors:** Yumei Feng, Chuang Wei, Xianzhang Zeng, Qianyun Pang, Zhangrong Xiong

**Affiliations:** Department of Anesthesiology, Chongqing University Cancer Hospital, Chongqing, China

**Keywords:** case, complications, CT-guided lung puncture, gas embolism, general anesthesia

## Abstract

**Background:**

CT-guided percutaneous lung puncture is a minimally invasive technique widely used for localizing pulmonary nodules prior to resection. While rare, systemic gas embolism represents a potentially catastrophic complication. This case series analyzes three patients undergoing general anesthesia for small lung lesion resection after CT-guided localization, aiming to improve their perioperative recognition and intervention.

**Case presentation:**

Case 1 experienced intraoperative cardiac arrest due to gas embolism but recovered fully after prompt cardiopulmonary resuscitation (CPR) and delayed thoracoscopic surgery. Case 2 developed postoperative hemiplegia and dysarthria due to delayed intervention, with partial neurological recovery after one month. Case 3 received immediate treatment, resulting in stable vital signs and no sequelae.

**Conclusion:**

These cases illustrate that CT-guided percutaneous lung puncture under general anesthesia carries both benefits and risks. Gas embolism may occur during the biopsy procedure, and if not promptly detected under general anesthesia, it could potentially lead to cardiac arrest. Hybrid operating rooms general anesthesia helps streamline workflow but may mask early signs of air embolism and pose specific risks. Key prevention and management strategies are extremely important, including routine single-lung isolation with the use of a double-lumen endotracheal tube or bronchial blocker, keeping the operated lung at low continuous positive airway pressure (CPAP) during puncture with minimal needle passage, and CT surveillance immediately after surgery. Multidisciplinary coordination, vigilant monitoring, and prompt intervention, including repositioning of the patient and administration of 100% oxygen, are essential to mitigate serious outcomes.

## Background

With advances in early diagnosis and treatment of lung cancer and the widespread adoption of low-dose computed tomography (LDCT), CT-guided percutaneous lung localization has become a key technique for the precise marking of small, deep, or radiologically occult lesions before video-assisted thoracoscopic surgery (VATS). It provides surgeons with visual or tactile guidance during VATS procedures, enabling rapid diagnosis and treatment of pulmonary nodules while avoiding the need for conversion to open surgery due to localization failure or excessive resection (1–3). The growing availability of hybrid operating rooms has further promoted an integrated “one-stop” diagnostic and therapeutic approach, enabling seamless transition from localization under general anesthesia to surgical resection (4, 5). This model mitigates the risks and logistical delays associated with patient transfer following conventional local anesthesia-based localization performed in standard CT suites. Studies indicate that approximately 53% of patients experience moderate-to-severe pain during localization under local anesthesia (6). Performing the procedure under general anesthesia significantly alleviates anxiety and discomfort, reduces motion-related complications, and improves targeting accuracy. Although minimally invasive and efficient, the technique carries risks such as pneumothorax, hemorrhage, and gas embolism. The latter, though rare, can lead to myocardial infarction, stroke, or even death (7). Sometime it will cause huge economic losses, data from the American Society of Anesthesiologists Closed Claims Project showed 100% of claims for gas embolism resulted in a median payment of $325,000 (8). The reported incidence of clinically apparent gas embolism ranges from 0.061 to 0.49%, while the asymptomatic incidence may be as high as 3.8–4.8% (7, 9–11). Due to its often subtle or absent clinical presentation, this complication is often overlooked. This article presents three cases of gas embolism occurring during CT-guided lung localization under general anesthesia, aiming to enhance clinical recognition and management of this serious complication and to improve patient outcomes.

## Case 1

### General information

**Figure 1:**
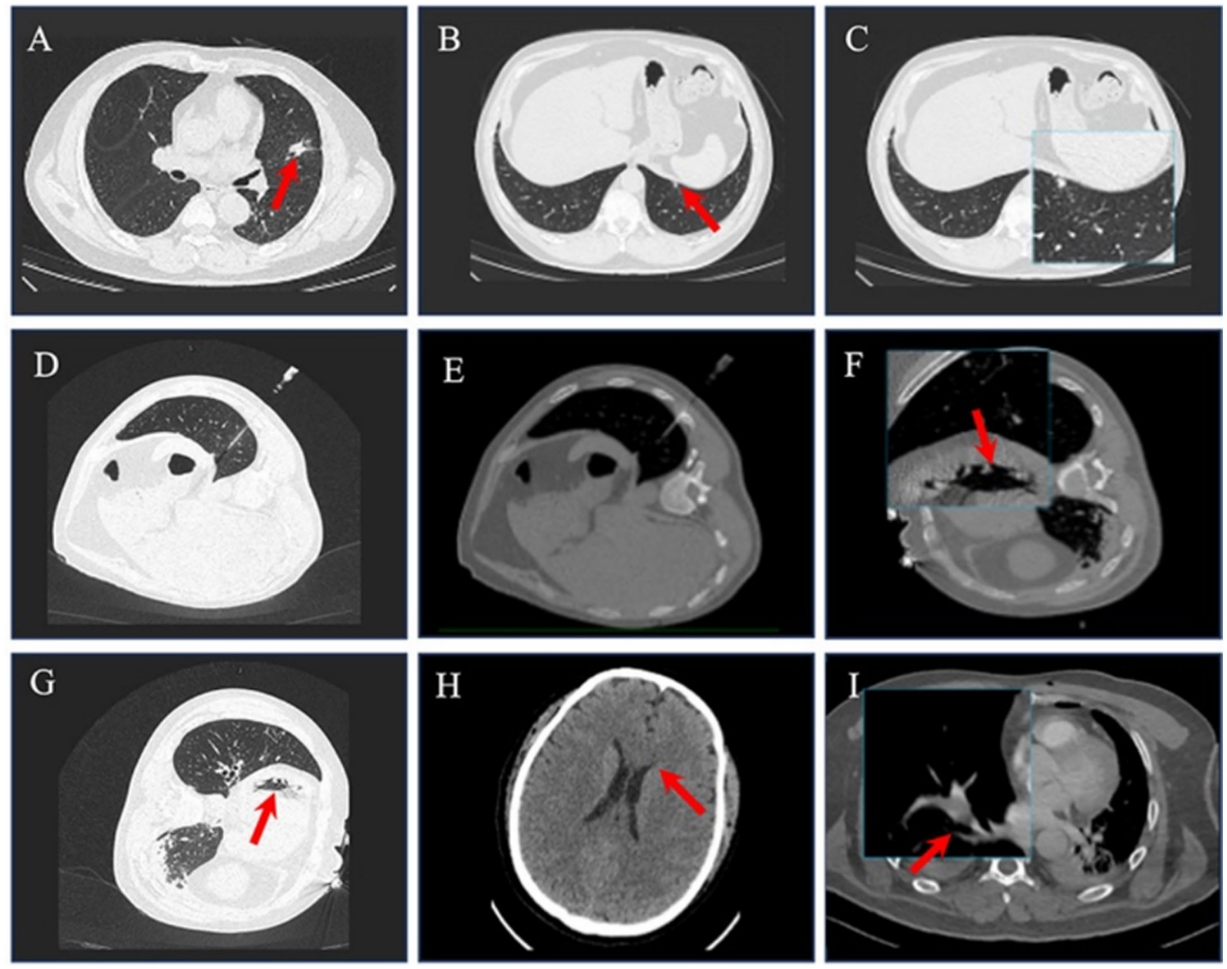
CT image of case 1. **(A)** CT scan a 17 × 13 mm spiculated nodule in the left upper lingular segment (red arrow), with suspected neoplastic features. **(B,C)** A 5 × 4 mm ground-glass opacity (red arrow and image magnification area) near the left diaphragmatic dome. **(D,E)** Location of the puncture needle during percutaneous pulmonary puncture. **(F,G)** Cardiac gas embolism following puncture (red arrow). **(H)** Head CT scan suspicious cerebral parenchymal edema (red arrow) after POD 6. **(I)** Pulmonary CTA scan embolus formation in the right middle lobe artery after POD 9.

A 54-year-old male (BMI 30.1 kg/m^2^, ASA II, NYHA II) presented with a 1^−^month history of progressive chest tightness and pleuritic pain. His medical history included right ureteroscopic lithotripsy under general anesthesia one year prior, with no documented perioperative complications. Social history revealed occasional ethanol use (<20 g/week) and lifelong tobacco abstinence. Preoperative blood tests, biochemical analyses, and electrocardiograms were normal except for a platelet count of 93 × 10^9/L. High-resolution chest CT (Figure 1) revealed two distinct pulmonary lesions:(1) A 17 × 13 mm spiculated nodule in the left upper lingular segment (Figure 1A). (2) A 5 × 4 mm ground-glass opacity near the left diaphragmatic dome (Figures 1B,C).

### Operative course and critical situations

The patient intends to undergo a combined surgery under general anesthesia, consisting of CT-guided fiducial placement in the left lower lobe lesion followed by immediate single-port video-assisted thoracoscopic surgery (VATS) for lingular segmentectomy (left upper lobe) and wedge resection (left lower lobe). After arriving in operating room, standard ASA monitors applied, followed by invasive hemodynamic monitoring via right radial artery and right internal jugular vein central line under ultrasound guidance using lidocaine 1% local infiltration. Standard rapid-sequence anesthetic induction was performed with propofol 2 mg/kg, rocuronium 0.9 mg/kg, and sufentanil 0.4 μg/kg. A 37-Fr right-sided double-lumen endotracheal tube was successfully inserted and positioned at 30 cm from incisors, confirmed by fiberoptic bronchoscopy. Initial ventilation parameters: volume-controlled mode with FiO_2_ 1.0, tidal volume 5–6 mL/kg predicted body weight (PBW), respiratory rate 14-16/min, PEEP 5 cmH_2_O, achieving ETCO_2_ 35–45 mmHg. The patient’s vital signs were stable: HR 80–90 beats/min, BP 120–150/65–90 mmHg, SpO2 99–100%.

The patient was positioned in the right lateral decubitus position. CT-guided planning confirmed the trajectory for accessing the left lower lobe nodule. A disposable pulmonary nodule localization needle (Ningbo Shengjiegang Biotechnology Co., LTD., model specification: SS510-10) was inserted percutaneously into the left lower pulmonary nodule during routine surgical disinfection. About 10 minutes later, the patient’s vital signs changed dramatically, showing BP 42/32 mmHg (invasive arterial), HR 70 bpm (sinus rhythm), ETCO₂ 24 mmHg, and SpO₂ 100% (pulse oximetry). Emergency management was performed by the anesthesiologist, which included administering two doses of 6 mg IV bolus ephedrine, rapidly repositioning the patient to a supine position, and adjusting ventilation to right one-lung ventilation (FiO₂ 1.0).

Ten minutes later, ECG demonstrated ventricular fibrillation. Advanced Cardiac Life Support protocol was initiated, which involved chest compressions, administration of epinephrine 1 mg IV every 3 min, defibrillation ×3 (200 J biphasic), ice cap-induced hypothermic brain protection, and continuous intravenous infusion of epinephrine (1 ug/kg/min) and norepinephrine (0.3 ug/kg/min). Echocardiography detected a few small spots and slightly hyperintense echoes in the left ventricle. One hours later, return of spontaneous circulation (ROSC) was achieved.

### Postoperative recovery

The patient demonstrated a gradual neurological recovery in the surgical ICU, achieving full consciousness (Glasgow Coma Scale 15) by the first postoperative day (POD 1). Extubation was successfully performed on POD 2. Vital signs remained stable post-extubation. Neuroimaging on POD 6 (Figure 1H) revealed: (1) Diffuse cerebral edema (Grey-White Matter Ratio 1.2, with the normal range being 1.3–1.4); (2) Sulcal effacement in the parieto-occipital regions; (3) Mild bilateral ventricular compression. Surveillance pulmonary CTA on POD 9 (Figure 1I) detected the formation of emboli in the right middle lobar artery, prompting the initiation of enoxaparin calcium anticoagulant therapy.

### Definitive surgical intervention

Following cardiopulmonary optimization, repeat single-port VATS was performed on POD 19, involving anatomical lingular segmentectomy (left upper lobe) and stapled wedge resection (left lower lobe). The operation was successful, postoperative recovery was satisfactory, and the patient was discharged successfully 12 days after surgery.

## Case 2

### General information

**Figure 2:**
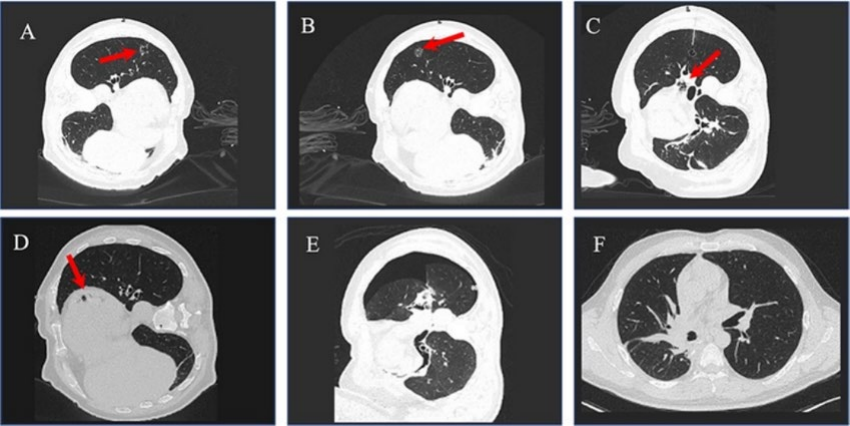
CT image of case 2. **(A)** Chest CT showed air-containing cavity in the posterior apical segment of left superior lung (red arrow), about 10 × 8 mm. **(B)** Contrast-enhanced chest CT scan a partially solid nodules in the left upper lobe (red arrow), about 21 × 16 mm. **(C)** Left pulmonary vein gas embolism (red arrow) at the time of puncture. **(D)** Left atrial gas embolism (red arrow); **(E)** Chest CT scan left pneumothorax after surgery. **(F)** Chest CT reexamination scan complete resolution of pneumothorax (no residual air/fluid) after POD 7.

A 66-year-old male (BMI 21.8 kg/m^2^, ASA II, NYHA II) presented with incidentally detected pulmonary nodules during routine surveillance. The patient had a 40-pack-year smoking history (ceased 4 years preoperatively) and consumed ethanol (<10 g/week). Preoperative blood gas analysis revealed PO_2_ 78 mmHg and PCO_2_ 53 mmHg, with no significant abnormalities noted in other laboratory tests. Contrast-enhanced chest CT identified a balloon-like space in the posterior segment of the upper lobe tip of the left lung (Figure 2A), measuring approximately 10 × 8 mm, and partial solid nodules in the left upper lobe (Figure 2B), measuring about 21 × 16 mm. Both lungs exhibited scattered chronic inflammation and pulmonary embolism in the anterior segment of the left upper lobe. Preoperative enoxaparin 4,000 IU SC q12h was administered for 5 days, discontinued 24 h preoperatively.

### Operative course and complication development

The patient intends to undergo a combined surgery under general anesthesia with double-lumen bronchial intubation, consisting of CT-guided percutaneous lung puncture localization followed by immediate single-port video-assisted thoracoscopic surgery (VATS) for lingular segmentectomy (left upper lobe) and wedge resection of apical-posterior lesion. Mixed hollow oxygen ventilation replaced pure oxygen dual-lung ventilation during the puncture process. Due to the difficulty of puncture, the surgeon repeatedly adjusted the puncture needle (total 4 passes). About 10 min after multiple puncture attempts, the interventional radiologist identified left pulmonary vein gas embolism and left atrial gas embolism (Figures 2C,D). Notably, hemodynamic stability persisted (MAP 65–75 mmHg). Pure oxygen single-lung ventilation was immediately initiated post-gas embolization. Given the stability of the patient’s condition, the decision was made to proceed with the operation, which lasted 3 hours and was completed successfully. Final intraoperative findings: (1) Frozen pathology: Invasive adenocarcinoma (pT1b) in lingula, metastatic focus in apical lesion; (2) Post-resection CT: Complete resolution of intravascular air, residual pneumothorax (Figure 2E).

### Postoperative course and recovery

The patient was extubated at 120 min post-op with stable vitals (SpO_2_ 96% on room air), anterior chest discomfort, clear consciousness, grade 0 muscle strength in the left upper and lower limbs, and speech stuttering. The patient was transferred to the ICU for further treatment, including anticoagulation and pulmonary care for one week, without specific examination or treatment for cerebral sequelae. Imaging Surveillance: (1) POD 7 CT (Figure 2F): Complete resolution of pneumothorax (no residual air/fluid). The patient was discharged with grade 0 left muscle strength and improved speech. (2) POD 45 Follow-up: The patient was able to walk, exhibiting grade 4 muscle strength in the left limb and normal speech. Neurological recovery: MRC 4/5 for the left limbs, NIHSS score of 0.

## Case 3

### General information

**Figure 3:**
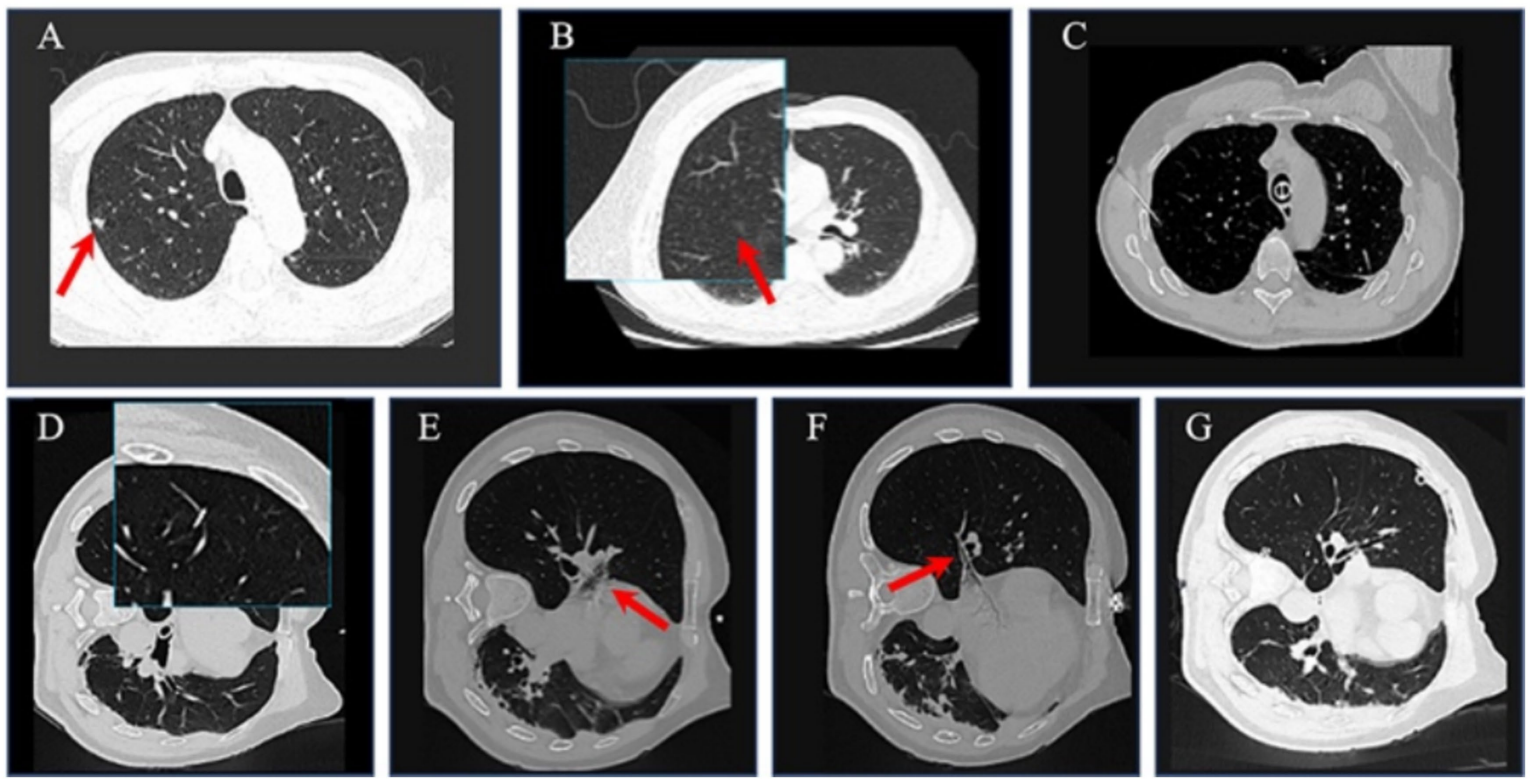
CT image of case 3. **(A)** Contrast-enhanced chest CT scan an 8 × 6 mm spiculated solid nodule (red arrow) with mild contrast enhancement in the apical segment of the right upper lobe. **(B)** Contrast-enhanced chest CT scan 5 × 4 mm ground-glass opacity nodule (red arrow) in the posterior segment of the right upper lobe. **(C)** Puncture of the right upper lobe apex. **(D)** Posterior branch of vein injured by puncture of ground glass tubercle of posterior upper lobe of right lung. **(E)** Right superior lobe venous gas embolism (red arrow). **(F)** Right lower lobe venous gas embolism (red arrow). **(G)** Postoperative whole chest CT scan showed disappearance of gas embolism in the right pulmonary vein [compared with **(E,F)** image during intraoperative puncture].

A 64-year-old male patient (BMI 20.59 kg/m^2^, ASA II, NYHA II) was referred to our institution for surgical evaluation of incidentally discovered pulmonary nodules detected during routine health screening 3 months prior. The patient reported a 50-year smoking history with a daily consumption of approximately 20 cigarettes, while denying alcohol use. Arterial blood gas analysis on room air showed PaO₂ 78 mmHg. Standard laboratory tests were normal. Contrast-enhanced chest CT imaging identified two distinct pulmonary lesions: (1) An 8 × 6 mm spiculated solid nodule with mild contrast enhancement in the apical segment of the right upper lobe (Figure 3A), and (2) A 5 × 4 mm ground-glass opacity nodule in the posterior segment of the right upper lobe (Figure 3B). Additional findings included panlobular emphysema and bilateral scattered inflammatory changes.

### Operative course and complication development

The patient underwent combined CT-guided percutaneous transthoracic needle aspiration marking followed by uniportal video-assisted thoracoscopic surgical (VATS) resection of the right upper lobe lesions under general anesthesia with double-lumen bronchial intubation, and was given mechanical ventilation with pure oxygen at 2 L/min. Two nodules were localized via intraoperative puncture (Figures 3C,D), and the puncture process proceeded smoothly. Following the puncture, the radiologist immediately performed the whole chest CT scan, and found that the patient developed right pulmonary venous gas embolism (Figures 3E,F). The anesthesia team implemented emergency protocols including immediate conversion to left one-lung ventilation (FiO₂ 1.0), Trendelenburg positioning, and hemodynamic optimization with phenylephrine infusion (0.1–0.3 μg/kg/min) to maintain MAP >65 mmHg. Despite these complications, the surgical team proceeded with uniportal VATS resection after under careful monitoring.

### Postoperative course and recovery

Repeat CT angiography demonstrated complete resolution of the pulmonary vein gas embolism (Figure 3G). The patient recovered from anesthesia with stable vital signs, clear consciousness, and no special discomfort approximately 10 min postoperatively, and discharged successfully at postoperative 7 days.

## Discussion

Systemic gas embolism represents a rare but serious complication of CT-guided percutaneous lung puncture localization. Its incidence rate in imaging diagnosis is significantly higher than that in clinical symptom diagnosis (4.8% vs. 0.17%) (10), and it may lead to catastrophic outcomes such as acute ischemic stroke, myocardial infarction, or death. The mechanism of systemic gas embolism following percutaneous lung puncture involves gas entering the pulmonary veins, passing through the left atrium and left ventricle, and ultimately entering the systemic circulation. Three principal pathways have been proposed for air entry into the pulmonary vein (12): (1) direct introduction through the puncture needle; (2) concurrent alveolar and venous penetration with pressure-driven gas influx; and (3) retrograde passage from the pulmonary artery to the pulmonary vein through pulmonary microcirculation or pre-existing arteriovenous malformations. The most dangerous consequence is when gas enters the coronary arteries and cerebral blood vessels: coronary events manifest as acute ischemia (chest pain, syncope, cardiac arrest), while cerebral involvement causes neurological deficits (sensory loss, aphasia, seizures). Embolization to other vascular territories can lead to hemodynamic instability, urinary retention, respiratory distress, or hemoptysis. In all three cases presented here, inadvertent injury to pulmonary veins adjacent to the target lesion could not be excluded as a contributing factor.

Proactive prevention remains essential. Key measures include ensuring the target lesion lies below the left atrial level, utilizing breath-hold techniques during needle placement, avoiding vascular structures and minimizing puncture attempts, and implementing post-procedural CT or MRI surveillance for early detection (13, 14). In the event of gas embolism, prompt intervention is essential to mitigate severe complications (15). Recommended measures include immediate repositioning of the patient into the supine or Trendelenburg position (with head lowered 15 to 30°) to facilitate air trapping in the right ventricle and prevent systemic embolization. Concurrently, administration of 100% oxygen is advised, as it enhances nitrogen washout and accelerates the elimination of air bubbles from the circulation. Hyperbaric oxygen therapy represents the most effective treatment for cerebral infarction resulting from air embolism, acting by increasing ambient pressure to reduce bubble volume and promote gas dissolution. This intervention should be initiated as early as possible (16, 17); however, it is contraindicated in patients with significant emphysema, bullae, or active pneumothorax.

Analysis of these three cases provides important insights into the advantages and challenges of CT-guided percutaneous lung localization under general anesthesia. Conducting the procedure in a hybrid operating room facilitates immediate resection following localization, eliminating delays associated with inter-departmental transfer and enhancing workflow efficiency. General anesthesia minimizes patient anxiety and pain, thereby reducing risks of needle displacement or repeated punctures. In cases of gas embolism, contralateral one-lung ventilation can be initiated promptly to limit systemic gas entry, while positional adjustments such as the Trendelenburg position help redirect intracardiac air. The fully equipped operating environment also enables timely resuscitation in the event of severe complications such as arrhythmia, hypotension, or cardiac arrest. However, we also identified shortcomings in handling these three cases: Regarding Case 1, due to a lack of timely communication with the surgeon and radiologist, we only recognized the occurrence of gas embolism when the patient developed severe hypotension during surgical sterilization and rapidly progressed to cardiac arrest requiring resuscitation. Case 2: Repeated puncture increased the risk of complications such as bleeding and air embolism although left ventricular gas embolism was detected promptly, but the patient’s stable vital signs during general anesthesia led to the oversight that gas had entered the cerebral circulation. The patient’s position was not adjusted when gas embolism occurred, and hyperbaric oxygen therapy was not administered postoperatively. Consequently, the patient developed sequelae of cerebral embolism. In contrast, Case 3 utilized a double-lumen tube for selective ventilation, enabling prompt intervention and an uneventful recovery.

In conclusion, highlights the critical role of meticulous surgical techniques, close radiological monitoring, and timely anesthetic intervention in reducing the risk of gas embolism and related sequelae. First, CT-guided percutaneous lung puncture localization may lead to serious complications such as air embolism. Currently, preoperative auxiliary localization techniques for pulmonary small nodules continue to evolve, with the increasing adoption of robotic bronchoscopy and electromagnetic navigation bronchoscopy. These bronchoscopic approaches avoid crossing the pleura and lung parenchyma, reducing the possibility of direct vascular injury and thereby lowering the incidence of pneumothorax and systemic air embolism. However, these techniques still have limitations such as high cost, operational complexity, time consumption, a steep learning curve, and reliance on multidisciplinary collaboration (18). Therefore, it is recommended that medical institutions select appropriate preoperative localization methods based on their specific conditions to reduce postoperative complications. Regardless of the localization technique used, it must be performed by an experienced surgeon with proficient skills. In the case of CT-guided percutaneous lung puncture localization, repeated punctures should be avoided. For example, in Case 2, four punctures were required to successfully mark the lesion, which undoubtedly increased the risk of bleeding and air embolism. In our institution, the current basic approach adopted is to use disposable pulmonary nodule localization needles for percutaneous lung puncture positioning under CT guidance for lung puncture positioning. The average number of punctures for successful localization is typically 1–3, but the actual number depends on nodule characteristics (size, location, depth) and operator experience. Secondly, effective interdisciplinary communication is essential. Strengthening collaboration among surgeons, radiologists, and anesthesiologists facilitates earlier detection and management of gas embolism. Finally, with the growing use of hybrid operating rooms, general anesthesia is increasingly preferred due to its workflow efficiency and enhanced patient comfort. Under local anesthesia, patients breathe room air, in which the high nitrogen content delays gas absorption. In contrast, patients under general anesthesia receive pure oxygen throughout the procedure. In the event of embolism, immediate initiation of single-lung ventilation, combined with absorption of pure oxygen, may help reduce postoperative complications (it is noteworthy that in Case 2, the patient did not receive pure oxygen after intubation). Although general anesthesia with tracheal intubation has been identified as a potential risk factor for systemic gas embolism (19)—because positive-pressure ventilation may facilitate gas entry into injured pulmonary veins—high-quality evidence directly comparing general and local anesthesia in this context remains limited. Based on current experience, we recommend that during needle localization under general anesthesia, a double-lumen endotracheal tube or bronchial blocker should be routinely used, accompanied by ventilation with 100% oxygen. Specifically, after airway fixation and lung preoxygenation, one-lung ventilation is maintained on the non-operative side during puncture, while a continuous positive airway pressure (CPAP) of 5 cmH₂O with 100% oxygen is applied to the operative lung. This strategy not only maintains lung expansion but also ensures a low and stable intrapulmonary pressure, significantly lower than pulmonary venous pressure. Theoretically, this pressure gradient helps reduce the risk of a large volume of gas entering the systemic circulation during venous puncture. Since implementing this protocol, no severe gas embolism events have been observed in our institution, though its efficacy requires further validation.

## Conclusion

Although rare, gas embolism secondary to CT-guided percutaneous lung biopsy can be fatal. While general anesthesia facilitates the procedure, it may also introduce specific risks, necessitating further investigation to fully delineate its safety profile. Based on our experience, the following measures are critical for enhancing safety under general anesthesia: Initiate 100% oxygen ventilation following intubation, maintain single-lung ventilation on the non-operative side during puncture, and apply continuous positive airway pressure (CPAP) at 5 cmH₂O to the operative lung. Minimize the number of puncture attempts to reduce tissue and vascular injury. Perform immediate post-procedural CT imaging in close collaboration with the radiology team to enable early detection of intravascular air. Upon suspicion or confirmation of embolism, promptly reposition the patient (e.g., Trendelenburg) to limit further gas entry into the systemic circulation. In summary, whether under general or local anesthesia, the consistent identification of high-risk scenarios, proactive prevention, and timely intervention remain essential to mitigating complications during CT-guided lung localization.

## Data Availability

The original contributions presented in the study are included in the article/supplementary material, further inquiries can be directed to the corresponding author.

## Glossary

CT: Computed tomography
CPR: Cardiopulmonary resuscitation
BMI: Body Mass Index
ASA: American Society of Anesthesiologists
NYHA: New York Heart Association
VATS: Video-assisted thoracoscopic surgery
FiO₂: Fraction of inspired oxygen
PBW: Predicted body weight
PEEP: Positive END-EXPIRATORY PRESSURE
ETCO₂: End-tidal carbon dioxide
HR: Heart rate
BP: Blood pressure
SpO₂: Peripheral capillary oxygen saturation
IV: Intravenous
ECG: Electrocardiogram
ROSC: Return of spontaneous circulation
ICU: Intensive care unit
POD: Postoperative day
CTA: Computed tomography angiography
PO₂: Partial pressure of oxygen
PCO₂: Partial pressure of carbon dioxide
IU: International unit
SC: Subcutaneous
MAP: Mean arterial pressure
pT1b: Pathological stage T1b
MRC: Medical Research Council
NIHSS: National Institutes of Health Stroke Scale
MRI: Magnetic resonance imaging
CPAP: Continuous positive airway pressure
